# A Role for Periostin Pathological Variants and Their Interaction with HSP70-1a in Promoting Pancreatic Cancer Progression and Chemoresistance

**DOI:** 10.3390/ijms252313205

**Published:** 2024-12-08

**Authors:** Yasuo Tsunetoshi, Fumihiro Sanada, Yuko Kanemoto, Kana Shibata, Atsushi Masamune, Yoshiaki Taniyama, Koichi Yamamoto, Ryuichi Morishita

**Affiliations:** 1 Department of Geriatric and General Medicine, Graduate School of Medicine, Osaka University, Suita 565-0871, Japan; tsunetoshi@geriat.med.osaka-u.ac.jp (Y.T.); kyamamoto@geriat.med.osaka-u.ac.jp (K.Y.); 2 Department of Clinical Gene Therapy, Graduate School of Medicine, Osaka University, Suita 565-0871, Japan; morishit@cgt.med.osaka-u.ac.jp; 3 Department of Breast and Endocrine Surgery, Graduate School of Medicine, Osaka University, Suita 565-0871, Japan; ykanemoto@onsurg.med.osaka-u.ac.jp; 4 Department of Advanced Molecular Therapy, Graduate School of Medicine, Osaka University, Suita 565-0871, Japan; shibata@cgt.med.osaka-u.ac.jp (K.S.); taniyama@cgt.med.osaka-u.ac.jp (Y.T.); 5 Division of Gastroenterology, Graduate School of Medicine, Tohoku University, Sendai 980-8575, Japan; atsushi.masamune.d2@tohoku.ac.jp

**Keywords:** periostin, extracellular matrix protein, alternative splicing variants, pancreatic cancers

## Abstract

Pancreatic ductal adenocarcinoma (PDAC) characterized by an abundant cancer stroma is an aggressive malignancy with a poor prognosis. Periostin (Pn) is a key extracellular matrix (ECM) protein in various tumor progression. Previously, we described the role of Pn alternative splicing variants (ASVs) with specific functional features in breast cancer. Pn is known to associate with a chemoresistance of PDAC, but the functions of the Pn-ASVs remain largely unknown. In this study, we focused on physiological and pathological Pn-ASVs, and examined the characteristics of Pn-expressing cells and the difference in function of each ASV. We found that cancer-associated fibroblasts (CAFs) are a main source of Pn synthesis, which selectively secrete pathological Pn-ASVs with exon 21 both in mouse and human samples. RNA sequencing identified a gene signature of Pn-positive CAFs associated with ECM-related genes and chemokines, factors that shape the chemoresistance tumor microenvironment (TME). Additionally, only pathological Pn-ASVs interacted with heat shock protein 70-1a (HSP70-1a), leading to significant rescue of gemcitabine-induced PDAC apoptosis. In silico analysis revealed that the presence or absence of exon 21 changes the tertiary structure of Pn and the binding sites for HSP70-1a. Altogether, Pn-ASVs with exon 21 secreted from CAFs play a key role in supporting tumor growth by interacting with cancer cell-derived HSP70-1a, indicating that Pn-ASVs with exon 21 might be a potential therapeutic and diagnostic target in PDAC patients with rich stroma.

## 1. Introduction

Pancreatic cancer is a poor prognosis cancer, with a low 5-year survival rate of approximately 10% and is an increasing cause of cancer-related death [1]. The diagnosis is usually made at an advanced stage, as symptoms are frequently minimal in the early stages of the disease. The major histological type of pancreatic cancer is adenocarcinoma with the majority being pancreatic ductal adenocarcinoma (PDAC) of ductal epithelial origin, which accounts for more than 90% of all pancreatic malignancies [2]. PDAC is characterized by the presence of an abundant extracellular matrix protein (ECM), which reduces the efficacy of conventional cancer treatments and even new target therapies [3]. Cancer-associated fibroblasts (CAFs) in ECM have recently been focused on as promising targets for anti-cancer interventions [4]. However, the lack of specific markers for CAFs and elusive subtypes of CAFs have hindered successful clinical trials. Periostin (Pn), one of the ECM proteins secreted mainly by CAFs, is known to be associated to cancer progression, metastasis and chemoresistance in a variety of malignancies including PDAC. These facts suggests that an abundance of Pn-positive CAFs is associated with a poor prognosis in PDAC [5,6], with reports showing a positive correlation between Pn secretion and clinical stage [7]. We previously reported that alternative splicing variants (ASVs) with exon 21 in the Pn c-terminal region were highly expressed in breast cancer specimens, while Pn-ASVs lacking exon 17 and 21 were constantly expressed in a variety of healthy organs [8]. Thus, we intended to focus on Pn-ASVs, which is secreted from CAFs in PDAC, and investigated its distribution and functional relationship with cancer progression. Recent advances in molecular subtyping of pancreatic cancer have provided a variety of biomarker information, but the number of approved drugs is still limited, and new molecular biological analyses and treatments are needed. Pn-ASV is a novel therapeutic target because of its stromal origin and independence of the neoantigen from cancer cells.

In this study, an analysis of clinical samples identified that Pn was mainly secreted from CAFs in the stroma of PDAC and Pn-ASVs. Exon 21 was highly expressed and secreted from CAFs together with Pn-ASVs lacking exon 17 and 21. Pn-positive CAFs were located closely to tumor cells. In a syngeneic mouse PDAC model, RNA-sequence examination identified that the RNA signature of Pn-positive CAFs had higher expression of ECMs (collagen type I alpha 1 chain (Col1a1), collagen type I alpha 2 chain (Col1a2), tenascin (Tnc), fibrillin-1 (Fbn1) and α-smooth muscle actin (Acta2)), integrin, Tgf-β, Mmp, Wnt signaling molecules and several chemokines (Ccl2, Ccl7, Ccl11) as compared to Pn-negative CAFs. These data suggested the role of Pn-positive CAFs in chemoresistance tumor microenvironment (TME) development and the existence of a reciprocal signaling network between Pn-positive CAFs and tumor cells. Indeed, an in vitro co-culture experiment of CAFs and PDAC cells demonstrated that Pn-ASVs with exon 21 dimerized with heat shock protein 70-1a (HSPA1A) from cancer cells mediated Pn-induced chemoresistance in cancer cells. However, Pn-ASVs lacking exon 17 and 21 could not bind it. In silico-based tertiary structural modelling and an examination of predicted docking interactions of the various Pn-ASVs with HSPA1A proteins has been analyzed. Due to alternative splicing at the c-terminus, the n-terminal protein region encoded by exons 1–15 shows structural disparities even though the amino acid sequence is the same. This structural change was expected to result in differences of site in docking proteins HSPA1A, suggesting that the Pn-ASVs present at the c-terminus may have an impact on the function of the n-terminal side. All these data indicate that Pn-positive, especially Pn-ASVs with the exon 21-positive CAFs subset, locate close to cancer cells and contribute to cancer progression in PDAC. Pn-ASVs with exon 21 might be a potential therapeutic and diagnostic target in PDAC patients.

## 2. Results

### 2.1. Pn Expression in the Pancreatic Cancer and Normal Human Pancreas Tissues

**Figure 1:**
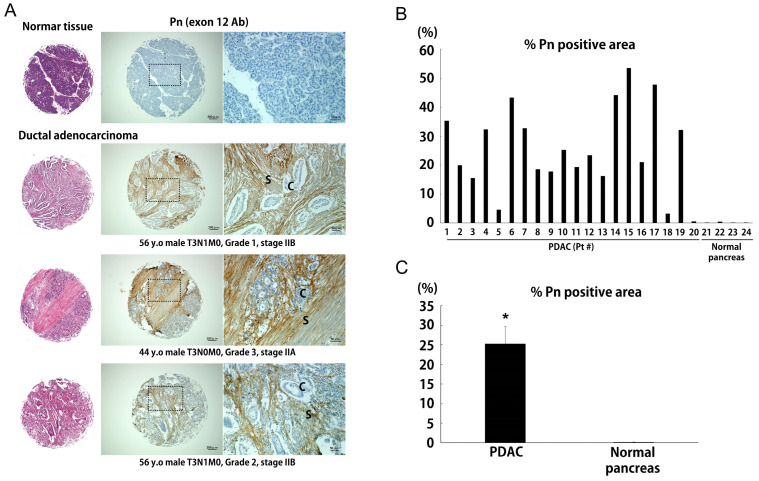
The expression of total Pn is elevated in PDAC. (**A**) Representative Pn immunohistochemical staining of normal pancreas and PDAC. S: stroma, C: cancer cells. (**B**) % of Pn-positive area in PDAC (n = 20) and normal pancreas (n = 4). (**C**) Average % of Pn-positive area in PDAC and normal pancreas. * *p* < 0.05 vs. normal pancreas.

To examine the localization of Pn protein in PDAC, tissue micro array (TMA) samples were analyzed by immunostaining using Pn antibody targeting exon 12, which theoretically recognizes all Pn-ASVs. In normal pancreatic tissue, the Pn-positive signal was negligible. On the other hand, strong staining was observed in the stroma of PDAC (Figure 1A) with limited staining in the parenchymal region (Figure 1A and Figure S1). In total, 17 out of 20 PDAC patients’ specimens had stained areas, >10%, whereas all of the healthy subjects had extremely low values (Figure 1B). PDAC was found to have significantly larger Pn-positive regions in the stroma as compared to healthy pancreas tissue (Figure 1C).

### 2.2. Pn ASVs Expression Pattern in CAFs

**Figure 2:**
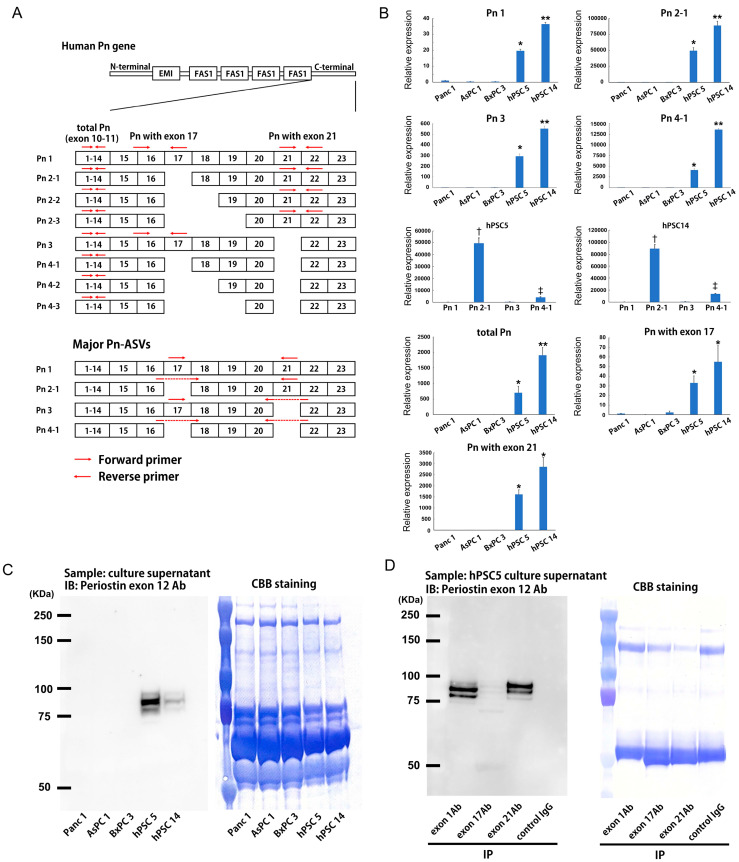
Pn-ASVs expression in PDAC and CAFs. (**A**) Human Pn-ASV structures. In addition to four major variants, Pn 1, Pn 2-1, Pn 3 and Pn 4-1, four other isoforms have been reported. Red arrows indicate the position of primers for Pn-ASV detection. The EMI domain, the four FAS-1 domains and the N- and C-terminal end of the carboxyl-terminal domain are depicted. (**B**) Pn-ASVs mRNA expression in PDAC cell lines (Panc 1, AsPC 1 and BxPC 3) and CAFs (hPSC5 and hPSC14). N = 4, * *p* < 0.05 vs. Panc 1, AsPC 1 and BxPC 3, ** *p* < 0.05 vs. Panc 1, AsPC 1, BxPC 3 and hPSC5. ^†^ *p* < 0.05 vs. Pn 1, Pn 3 and Pn 4, ^‡^ *p* < 0.05 vs. Pn 1 and Pn 2-1 and Pn 3. (**C**) Total Pn protein secreted from PDAC cell lines (Panc 1, AsPC 1 and BxPC 3) and CAFs (hPSC5 and hPSC14). (**D**) Pn-ASVs protein secreted from CAF. Pn-ASV proteins immunoprecipitated from a culture supernatant of CAFs using the specific antibodies against Pn exon 1, 17 and 21 were analyzed by Western blotting with Pn antibody for exon 12.

The expression of Pn-ASVs in PDAC cells and CAFs were examined by quantitative RT-PCR using PDAC cell strains, Panc1, AsPC1 and BxPC3, and CAFs strains, hPSC5 and hPSC14, derived from human pancreatic cancer. Previously, we referred to the major four Pn-ASVs with no exon splice-outs as Pn 1, those lacking exon 17 as Pn 2-1, those lacking exon 21 as Pn 3 and those lacking exon 17 and exon 21 as Pn 4-1 (Figure 2A). As shown in Figure 2B, the expression of all major Pn-ASVs were higher in CAFs compared to cancer cells, and the largest differences in relative expression was Pn 2-1. Measurement of relative expression of major Pn-ASVs in CAFs revealed that Pn 2-1 and Pn 4-1 mRNA expressions were significantly higher than Pn 1 and Pn 3. In addition to exon 17 and 21, exon 18 and 19 defects have recently been reported. Accordingly, Pn-ASVs with exon 17 or 21 were measured using primer sets shown in Figure 2A. The expression of both Pn with exon 17 or 21 were higher in CAFs than that in cancer cells, and the largest differences in relative expression was Pn-ASVs with exon 21 (Figure 2B). These data indicate that Pn-ASVs with exon 21 were predominantly synthesized in CAFs. Then, Pn-ASVs secreted from PDAC cells and CAFs were measured by antibodies specifically designed to recognize target exons. Using an antibody against exon 12, all Pn-ASV proteins secreted into the cell culture supernatant were analyzed and found to be predominant in CAFs (Figure 2C). Furthermore, the secretion of exon 1-, 17- and 21-containing variants in CAFs was detected by immunoprecipitation using antibodies specific for Pn exon 1, 17 and 21 (Figure 2D). Strong signals were observed in lanes of Pn-ASV protein precipitated with Pn 1 and 21 antibody. These results indicate that Pn is mainly synthesized and secreted from CAFs in vitro and that Pn-ASVs with exon 21 are predominantly secreted from CAFs than other variants.

### 2.3. Localization of Pn-Expressing Cells in PDAC

**Figure 3:**
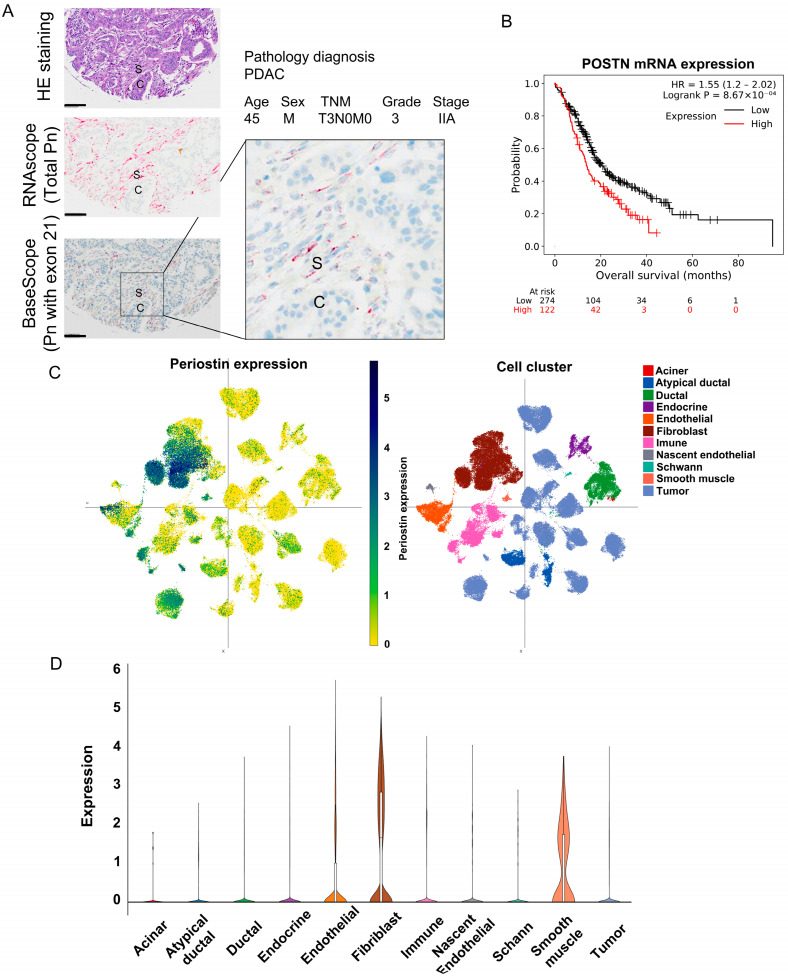
Restricted distribution of Pn-ASVs with exon 21 and sequencing analysis of Pn-positive cells. (**A**) A representative in situ hybridization image of total Pn (RNAscope) and Pn-ASVs with exon 21 (Basescope) in PDAC specimen. The red color indicates a positive signal for Pn mRNA. Both total Pn and Pn-ASVs with exon 21 mRNA was expressed in fibroblasts surrounding cancer. S: stroma, C: cancer cells. Bar indicates 100 μm. (**B**) Pn expression and prognosis in patients with PDAC. Pn expression and PDAC prognostic analysis was examined using the Kaplan–Meier plotter. It was found that the prognosis was worse in the group with high Pn expression. (**C**) Single-cell RNA-sequence analysis in different cell types show that the fibroblast and smooth muscle cell clusters are the ones that show high Pn expression. (**D**) Violin plot for Pn expression in different cell types in PDAC on the data from the Single Cell Portal.

Localization of Pn-expressing cells were examined in human PDAC TMAs by in situ hybridization. A probe targeting total Pn (n-terminal) and a probe designed to target exon 21 were used to examine mRNA localization. Both probes showed strong signals in the stroma, especially cells surrounding cancer cells (Figure 3A and Figure S2). These total Pn- or Pn exon 21-positive cells had spindle-shaped nuclei and elongated cell morphology. Since CAFs are spindle-shaped and Pn has been previously reported to be highly expressed in CAFs using single-cell RNA-seq, the spindle-shaped cells observed in this in situ hybridization were expected to be CAFs [9,10]. Indeed, single nucleus RNA-seq data of treatment-naïve PDAC on the Single Cell Portal demonstrated that Pn expression was found in clusters of fibroblasts and smooth muscle cell (Figure 3B,C). Additionally, when Pn expression and prognosis were examined using the Kaplan–Meier plotter, it was found that the prognosis was significantly worse in the group with Pn-high expression compared to the Pn-low group. The Pn expression patterns were similarly observed in head and neck cancers (Figure S3) and glioblastoma (Figure S4). Based on these results and previous reports [5,6], CAFs are thought to be the main source of cells producing Pn-ASVs with exon 21 in PDAC.

### 2.4. mRNA Expression Signature of Pn-Positive CAFs

**Figure 4:**
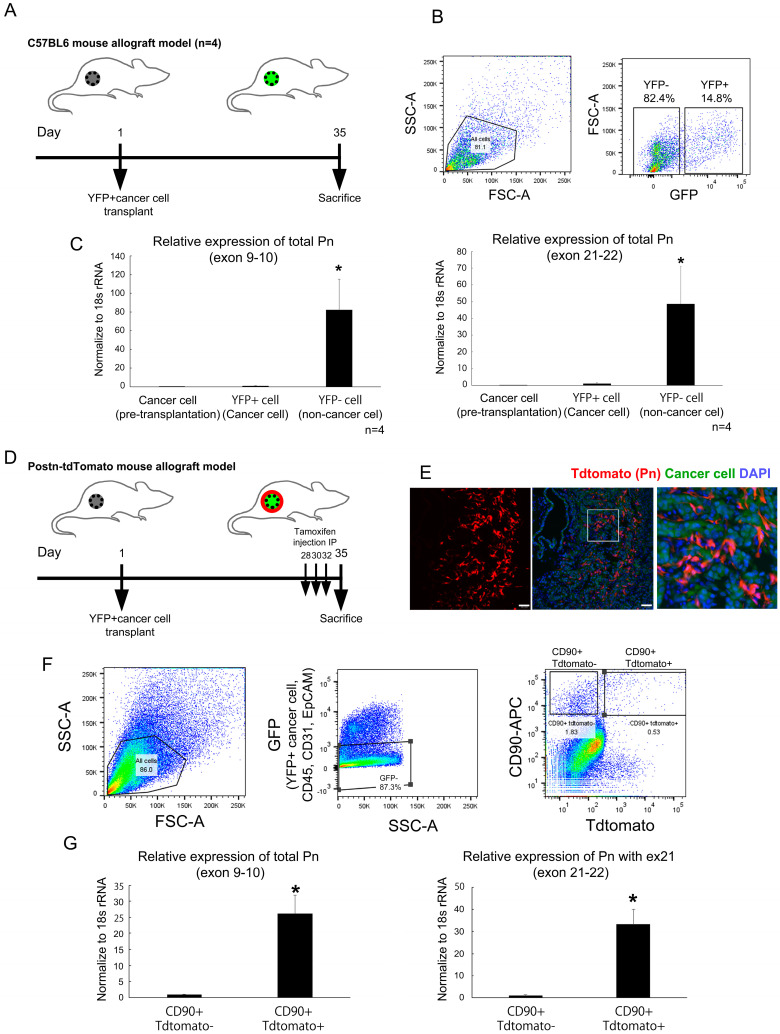
PDAC tumors activate Pn expression in CAFs. (**A**) KPC mice-derived YFP+ PDAC cells were subcutaneously implanted in the back of 8-week-old male C57BL6 mice and sacrificed on day 35. (**B**) YFP-positive and negative cells isolation by fluorescence-activated cell sorting (FACS). (**C**) Total Pn (amplicon: exon 9–10) and Pn-ASV with exon 21 (amplicon: exon 21–22) expression was higher in YFP-negative cells containing CAFs. N = 4, * *p* < 0.05 vs. pre-transplantation cancer cell and YFP+ cell. (**D**) YFP+ PDAC cells were subcutaneously implanted into the back of 8-week-old male Postn-tdTomato mice and sacrificed on day 35. Tamoxifen was administered intraperitoneally 5 days prior to sacrifice. (**E**) Tdtomato, a Pn-positive signal, was identified in the stroma of the excised tumor. TFP-positive ODAC cells were shown in green. White bar indicates 100 μm. (**F**) CAFs were isolated from excised tumors by FACS using GFP-negative and CD90-positive sorting. Tdtomato was further used to divide the CAFs to Pn-positive and negative groups. (**G**) Tdtomato-positive CAFs had higher expression of total Pn (exon 9–10 amplicon) and exon 21-containing ASVs (exon 21–22 amplicon). N = 3, * *p* < 0.05 vs. CD90+ Tdtomato-CAFs.

**Figure 5:**
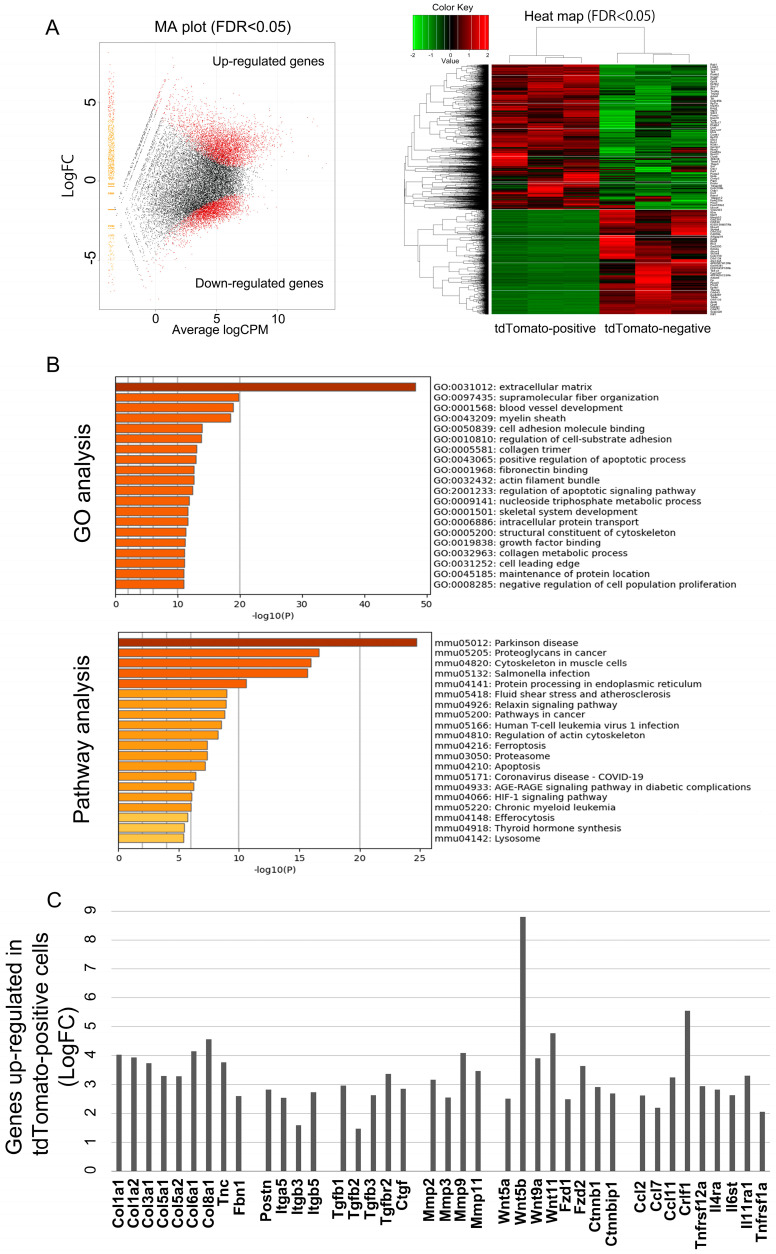
The transcriptome of Pn-positive (CD90+ tdTomato+) and negative (CD90+ tdTomato-) CAFs. RNA sequence analysis was performed using Pn-positive and Pn-negative CAFs isolated from a mouse PDAC syngeneic model. RNA-seq analysis comparing Pn-positive and Pn-negative CAFs. (**A**) RNA-seq analysis showing a volcano plot and heat map of differentially expressed genes (DEGs) in Pn-positive and Pn-negative CAFs. The data represent three biological replicates. It revealed 7624 differentially expressed genes (DEGs, red dots in MA plot) when comparing Pn-positive CAFs from Pn-negative CAFs (FDR < 0.05). Among 7624 DEGs, 4418 were up-regulated, while 3206 were up-regulated in Pn-positive CAFs. (**B**) Gene ontology enrichment and KEGG analysis of up-regulated genes in the Pn-positive CAFs from the RNA-seq data. (**C**) Representative genes whose expression was increased in the Pn-positive CAFs group. Up-regulated genes in Pn-positive CAFs include several genes related to cancer progression and chemoresistance. Data are the log2 of fold change (LogFC). Relative expression pattern analysis of up-regulated genes in Pn-positive CAFs by qRT-PCR analysis to validate the RNA-seq data is shown in Supplementary Figure S4.

To examine the function of Pn-expressing cells in vivo, yellow fluorescent protein (YFP)-positive mouse PDAC cells derived from the Kras^LSL-G12D^; p53^LoxP^; Pdx1-CreER triple mutant model of tamoxifen-inducible PDAC mice (KPC mice) were subcutaneously transplanted into 8-week-old wild-type male mice (Figure 4A). On day 35 after transplantation, tumors were excised, collected separately into YFP-positive and negative cells using flow cytometry (Figure 4B) and examined for Pn expression by quantitative reverse transcription polymerase chain reaction (RT-PCR). Expression of total Pn amplified by primer pares from exon 9 to 10 was significantly higher in the YFP-negative non-cancer cell population (Figure 4C). Then, the PDAC cells were subsequently transplanted into Pn-linage tracing mice (Figure 4D), and tandem dimeric Tomato (tdTomato)-positive cells were confirmed in the cancer stroma by tamoxifen administration (Figure 4E). The excised tumors were subjected to flow cytometry to remove epithelial cells, endothelial cells and hematopoietic cells using fluorescein isothiocyanate (FITC) conjugated Ep-CAM, CD31 and CD45 antibodies, respectively. This method also eliminated YFP-positive cancer cells by collecting FITC-negative population. The CD90-positive CAFs population was then collected separately into tdTomato-positive and negative fractions (Figure 4F). We confirmed that both total Pn and Pn-ASVs with exon 21 mRNA were predominantly expressed in CD90+ tdTomato-positive cells confirming the quality of the analysis method (Figure 4G). The transcriptome of CD90+ Pn-positive and CD90+ Pn-negative CAFs from a mouse PDAC syngeneic model was analyzed by RNA-sequencing. It revealed 7624 differentially expressed genes (DEGs) when comparing Pn-positive CAFs from Pn-negative CAFs (FDR < 0.05). Among these genes, 4418 were up-regulated while 3206 were down-regulated, highlighting a different transcriptional behavior of Pn-positive and negative CAFs (Figure 5A). GO term enrichment and Kyoto Encyclopedia of Genes and Genomes (KEGG) pathway analysis was used to explore biological processes, molecular functions, cellular components and pathways significantly enriched among the differentially expressed genes (DEGs) (false discovery rate (FDR) ≤ 0.001), focusing on those most relevant to cancer. As shown in Figure 5B, the most enriched GO category was extracellular metrics, reflected in the modulation of ECM of PDAC. Pathways associated with “proteoglycans in cancer” as well as “pathways in cancer” were up-regulated in Pn-positive CAFs with respect to Pn-negative CAFs. Among genes in the category “proteoglycans in cancer” were Col1a2, collagen type III alpha 1 chain (Col3a1), matrix metalloproteinase-2 (Mmp2) and Fn1, recapitulated in the chemoresistant ECM in PDAC, while the categories “pathways in cancer” included the genes Fgfr1, Bax, Cfl1, Calm1 and Cdk2, which are involved in cell survival, migration and proliferation (Figure S5). In addition, several integrins, which are known to be receptors for Pn, the expression of Tgf-β1 and its receptor, which induces Pn expression, and wnt, which is known to form a complex with Pn, were predominantly higher in the Pn-positive CAFs population (Figure 5C and Figure S6). These results suggest that Pn-positive CAF is a subtype of CAFs that form a cancer microenvironment that causes resistance to anti-cancer drug therapy, as has been previously reported for breast and head and neck cancers [11,12].

### 2.5. Pn-ASVs with Exon 21 Interacts with HSP70 and Promotes Gemcitabine Resistance in Pancreatic Cancer

**Figure 6:**
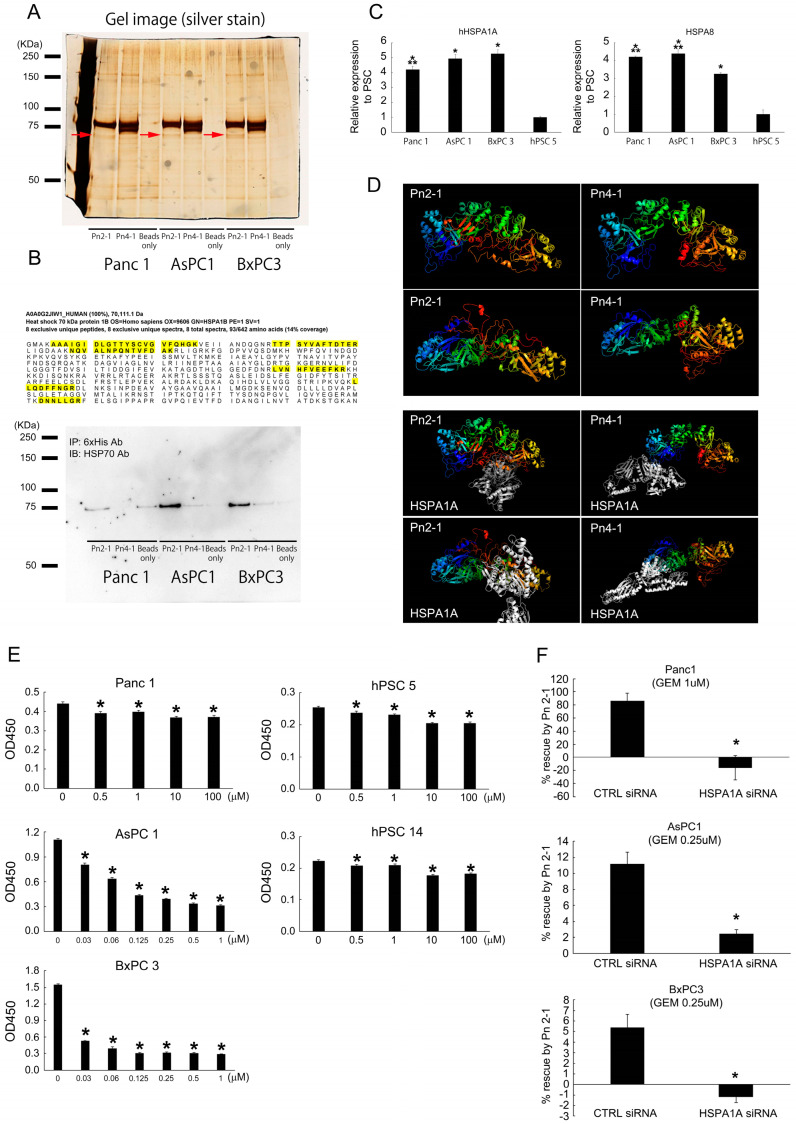
Pn-ASVs with exon 21 interacts with HSP70 and promotes gemcitabine resistance in pancreatic cancer. (**A**) Pull-down solutions were electrophoresed on gels and silver stained. Protein analysis of bands that appeared specifically in the pull-down solution of Pn2-1 was performed (red arrows). (**B**) Amino acid sequence of HSP70 was detected by LC-MS/MS analysis of gel bands analysis. Western blotting with primary antibody for HSP70 in post-pull-down solution detected a strong band in lane Pn2-1 but not Pn4-1. (**C**) HSPA1A and HSPA8 expression in PDAC cells and CAFs. HSP70 was higher in PDAC cell lines as compared to CAFs. N = 4, * *p* < 0.05 vs. hPSC 5, ** *p* < 0.05 vs. BxPC 3. (**D**) The 3D predicted steric structures of Pn2-1 and Pn4-1, and predicted binding sites with HSPA1A are shown. Colored blue to red from n-terminus to c-terminus. (**E**) GEM significantly reducing proliferation of PDAC and CAFs cell lines. N = 6, * *p* < 0.05 vs. 0 μM. (**F**) Pn-ASV with exon 21 suppresses GEM-induced PDAC cell line cell death. However, knockdown of HSPA1A prevents the rescue benefit. N = 12, * *p* < 0.05 vs. CTRL siRNA.

To search for proteins that interact with exon 21-containing Pn-ASVs secreted by CAFs, PDAC cell lines and CAFs were co-cultured, and the culture supernatant was pulled down with His tagged-recombinant Pn-ASV with exon 21 (Pn 2-1) or without exon 21 (Pn 4-1) (Figure S7). Gel electrophoresis of the pull-downed solution and silver staining revealed a dark band around 70 kDa in Pn 2-1 (Figure 6A). Liquid chromatography/mass spectrometry (LC-MS/MS) analysis of gel bands identified a heat shock protein 70 (HSP70) as a potential binding partner of Pn 2-1. Western blotting analysis of recombinant Pn pull-down samples with a primary antibody for HSP70 revealed a strong band in Pn 2-1 pull-downed samples compared to Pn 4-1 samples in all cancer cell lines, suggesting an interaction between Pn 2-1 and HSP70 (Figure 6B). The most frequently expressed subtypes of the HSP70 family, HSP70-1a (HSPA1A) and HSC70 (HSPA8), were examined for expression by quantitative RT-PCR. As shown in Figure 6C, their expression was higher in PDAC cell lines than CAFs. Since HSPA1A has recently been reported to be involved in anti-cancer drug resistance among the HSP70 family [13], we predicted the binding of Pn 2-1 to HSPA1A at in silico analysis. A protein data bank (PDB) file of the amino acid sequence was created using Phyre2, and each protein was visualized in Jmol. Then, protein–protein docking was predicted by ClusPro. As a result, HSPA1A was predicted to bind to the c-terminal side of the Pn 2-1 and to the n-terminal side of the Pn 4-1 (Figure 6D). Interestingly, the presence or absence of exon 21 not only altered the tertiary structure of the c-terminal side, but also the n-terminal side, although two variants have the same amino sequence at n-terminus (exon 1–15). These results suggest that differences in the splicing variants present in the Pn c-terminus may cause different protein-binding capacity and regulate the function of the binding proteins. The fact that Pn-ASVs with exon 21 interacts with HSPA1A suggests a functional role for HSPA1A in Pn signaling. We evaluated the gemcitabine (GEM) sensitivity of each PDAC cell line and CAFs and found that Panc1 and CAFs showed relatively GEM resistance compared to human PDAC cell lines, AsPC 1 and BxPC 3 (Figure 6E). These results are consistent with previous reports that CAFs are more resistant to anti-cancer drugs than tumor cells [14] and that anti-cancer drugs activate Pn signaling in CAFs [15]. Then, we examined the impact of HSPA1A knockdown on Pn-mediated anti-cancer drug resistance. Specific knockdown of HSPA1A by siRNA was confirmed in PDAC cell lines (Figure 6C) and change in % rescue by recombinant Pn 2-1 in GEM-treated PDAC cell lines was measured (Figure 6F). % rescue by recombinant Pn 2-1 was Panc1 86.3%, AsPC1 11.2% and BxPC3 5.4% in the CTRL siRNA group. However, the effect was counteracted significantly in the HSPA1A knockdown group. These results suggest that Pn-ASVs with exon 21 promotes GEM resistance in pancreatic cancer through HSPA1A. Therefore, Pn-ASVs with exon 21 might be a potential therapeutic and diagnostic target in PDAC patients, especially those who show chemoresistance.

## 3. Discussion

Pn is a nonstructural ECM protein highly secreted at sites of acute injury or chronic inflammation and promotes cell proliferation and fibrosis [16], but has also been found in normal tissues such as skin, lungs, stomach, colon, heart valves and placenta [8,17]. In malignant tumors including PDAC, Pn expression is also known to be enhanced, increasing tumor cell invasion and metastasis, eventually leading to chemoresistance [18,19,20]. On the other hand, it has been reported that epithelial-derived Pn functions as a tumor suppressor by stabilizing p53 and E-cadherin proteins via the Rb/E2F1/p14ARF/Mdm2 signaling pathway in gastric cancer [21]. Indeed, genetic knockdown of the total Pn in mice shows the deterioration of tumor growth and metastasis in several mouse xenograft models [22], suggesting that Pn may be involved in both tumor progression and inhibition. The Pn protein is composed of a functional n-terminal signal peptide required for secretion, a cysteine-rich region known as the EMI domain, a tandem of four repeating conserved fasciclin-like (FAS1) domains and a variable hydrophilic c-terminal domain with unknown function (Figure 2A). It has been demonstrated that the EMI domain directly binds to fibronectin and interacts with collagen, the four FAS1 domains interact with Tenascin-C, bone morphogenetic protein-1 (BMP-1) and cellular communication network factor 3 (CCN3), but the significance of the molecular function of the c-terminal of Pn-ASVs is not well understood [23,24]. A previous report shows that Pn mainly secreted in the stroma of tumor and exon 21-containing ASVs induces angiogenesis, tumor-associated macrophage polarization in triple-negative breast cancer (TNBC), cancer cell proliferation, invasion and metastasis in head and neck cancer [25,26], while exon 17- and exon 21-deficient ASVs are physiological subtypes and have limited function of angiogenesis [8,25]. PDAC is a lethal disease known for its dense tumor stroma. Thus, in this study, we examined *Pn*-ASVs expression in PDAC and whether exon 21-containing ASVs were involved in cancer progression. Immunostaining confirmed the expression of Pn in the stroma, and ISH similarly showed that exon 21-containing ASVs were localized in the stroma. Quantitative RT-PCR showed high expression of exon 21-containing ASVs and physiological variants in the CAFs cell line, which is negligible in PDAC cell lines, suggesting a main source of *Pn*-ASVs from CAFs. Additionally, two CAFs populations, *Pn*-positive and negative CAFs, were found to exist in PDAC tumors implanted in mice. RNA-sequencing of both populations revealed that *Pn*-positive CAFs expressed higher levels of integrin, *MMP-9*, *fibronectin* and *β-catenin*, several cytokines and chemokines than *Pn*-negative CAFs. These molecules are known to interact with Pn, stimulate downstream PI3K/Akt and nuclear factor kappa B (NF-kB) pathways (Figure 5C), leading to cancer cell survival, metastasis and anti-cancer drug resistance [27,28,29]. These data are consistent with the report that PDACs with more Pn-positive CAFs have a worse prognosis than those with more Pn-negative CAFs [5,30]. Experiments using PDAC cells and CAFs co-culture supernatants revealed that exon 21-containing Pn-ASVs interact with HSP70. The HSP70 family folds protein and controls protein quality by binding to exposed hydrophobic residues in an adenosine triphosphate (ATP)-dependent manner [13]. The majority of HSP70 is constitutively expressed mainly in the cytoplasm and nucleus, but HSPA1A expression increases under stress and extracellular HSPA1A has been identified in cancers [13,31]. Increasing evidence emphasizes extracellular HSPA1A as an important player in mediating immune responses in cancer by either exerting pro- or anti-tumorigenic functions. The assay system used in this study suggested that HSPA1A may be involved in anti-cancer drug resistance as a docking protein for Pn-ASVs with exon 21. However, there is still much unknown or controversially described; thus, specific functions and their associated mechanism need to be investigated. In silico-based tertiary structural modelling and an examination of predicted docking interactions of the various Pn-ASVs with HSPA1A proteins has been analyzed. Due to alternative splicing at the c-terminus, the protein region encoded by exons 1–15 (n-terminus) shows structural disparities even though the amino acid sequence was the same. This structural change of Pn-ASVs was expected to result in differences in the docking proteins site. Pn-ASVs with exon 17 has been reported to exhibit different binding patterns to TGF-β [32]. All these data suggest that the Pn-ASVs present at the c-terminus may influence the function of the n-terminal side. This study revealed that both pathological Pn-ASVs containing exon 21, which rarely express under physiological conditions but increase with pathogenesis, and physiological Pn-ASVs lacking exons 17 and 21 are predominantly expressed and secreted from CAFs in PDAC. The Pn-positive CAFs had a different genetic signature from the negative CAFs, indicating that they may be a more cancer-promoting CAFs population. Furthermore, pathological Pn-ASVs was predicted to bind with HSPA1A protein in different manner from physiological Pn-ASVs. These experimental results potentially suggest that pathological Pn-ASVs are a promising diagnostic stratification and therapeutic target to overcome the chemoresistance of stroma-rich PDAC. Whereas existing drugs target cancer cells, future studies may be able to improve therapeutic resistance by targeting Pn and other molecules in the stroma as well.

Finally, there are several limitations in this study. First, although the specific expression pattern of Pn-ASV with exon 21 was proved in this study, the recession of resistance to anticancer drug therapy by Pn21-ASV suppression has not been proven. Second, more studies with a larger number of specimens are needed to establish future stratified treatment. Third, CAFs were targeted in this study, but Pn-positive epithelial cells have also showed Pn expression in Pn-positive epithelial cells and smooth muscle cells in single-cell sequencing analysis, and the role of Pn-ASV in each cell has not been examined.

## 4. Materials and Methods

### 4.1. Tissue Micro Array

Pancreatic TMAs were obtained from US Biomax (Derwood, MD, USA). Immunohistochemical staining was performed using periostin antibody (AdipoGen, San Diego, CA, USA, cat. #AG-20B-0033-C100). To calculate the staining area within TMAs, we used the ImageJ software, version 1.53 program downloaded from the National Institutes of Health (Bethesda, MD, USA) website. The area of Pn-positive DAB-stained and HE-stained regions was calculated using ImageJ following HDAB color-deconvolution. The % of Pn positive area was calculated by dividing the Pn-positive area by the total tumor area.

### 4.2. Cell Lines and Cell Cultures

6422C1 KPCY mouse pancreatic cancer cell line (YFP-positive) was obtained from Kerafast (Shirley, MA, USA). This cell line is derived from LSL-KrasG12D/+; LSL-Trp53R172H/+; Pdx-1-Cre mouse. Panc-1 human PDAC cell line and hPSC5 and hPSC14 human pancreatic stellate cell lines derived from PDAC patients were from RIKEN BioResource Research Center (Ibaragi, Japan). AsPC-1/CMV-Luc and BxPC-3-Luc 2 human PDAC cell lines were purchased from JCRB (Osaka, Japan). 6422C1 was cultured in Dulbecco’s Modified Eagle Medium (DMEM) with 10% fetal bovine serum (FBS). Panc-1, AsPC-1 and BxPC-3 were cultured in RPMI1640 medium with 10% FBS. D-MEM/Ham’s F-12 medium with 10% FBS was used for hPSC5 and hPSC14 cell culture.

### 4.3. Quantitative RT PCR

Cellular total RNA was extracted according to protocol using the FastGene RNA Premium Kit (cat. #FG-81050, Nippon Genetics, Tokyo, Japan). cDNA was synthesized from total RNA using the High-Capacity cDNA Reverse Transcription Kit (cat. #4368814, Applied Biosystems, Waltham, MA, USA). Realtime PCR was performed in QuantStudio 7 using the primer sets shown in Table 1 with Fast SYBR Green Master Mix (cat. #4385612, Applied Biosystems). In each experiment, human 18SrRNA was amplified as a reference standard.

### 4.4. Western Blot Analysis

Cell culture supernatants were concentrated using Amicon Ultra-15 (UFC903024, Cambridge, MA, USA) for WB. Cell culture supernatant was electrophoresed and blotted onto poly vinyl dene fluoride (PVDF) membranes (cat. #LC2002, Invitrogen, Waltham, MA, USA). The membranes were incubated with the following primary antibodies and secondary antibodies for ECL analysis. Primary antibodies against periostin exon 1 (IBL, Gunma, Japan), exon 12 (AdipoGen, CA, USA, cat. #AG-20B-0033-C100) and secondary anti-mouse IgG, HRP-linked antibody (cat. #7076, Cell Signaling Technology, Danvers MA, USA) were used. Anti-periostin exon 17 and exon 21 antibodies were established using human periostin exon 17 and 21 peptides.

### 4.5. Co-Culture and His-Tag Pulldown

PDAC and CAF were co-seeded 1.5 × 10^5^ each cell in 6-well plate dishes and incubated in serum-free medium for 72 h. Collected culture supernatant and His-tagged recombinant periostin–beads (Dynabeads His-Tag Isolation and Pulldown kit, cat. #DB10103, Thermo Fisher, Waltham, MA, USA) complex were incubated, then pull-downed with a magnet and eluted with elution buffer (Figure S7). CJ39 periostin/OSF-2 (cat. #Q15063, Bon Opus Biosciences, Millburn, NJ, USA) was used as recombinant Pn 2, and recombinant human periostin protein HPLC-verified (cat. #10299-H08H, SinoBiological, Beijing, China) was used for recombinant Pn 4. The eluate was electrophoresed on a gel and silver stained (2D-Silver Stain Reagent II, cat. #423413, CosmoBio, Tokyo, Japan) for gel bands LC-MS/MS analysis in Osaka University omics center.

### 4.6. Knockdown by siRNA

Cells were seeded 1.0 × 10^5^ in 12-well plate dishes and replaced with a cocktail of siRNA, Lipofectamin 2000 (Cat. #11668027, Life Technologies, Carlsbad, CA, USA) in OPTI-MEM (Cat. #31985062, Life Technologies, Carlsbad, CA, USA) on day 2. From day 3, cells were incubated with or without gemcitabine (Cat. #170009582, NK, Tokyo, Japan) in serum free medium for 48 h. Control siRNA-A (Cat. #SC-37007, Santa Cruz, Dallas, TX, USA) and HSPA1A siRNA, Human (Cat #SC-29352, Santa Cruz) were used.

### 4.7. In Situ Hybridization and Single Nucleus RNA-Seq Analysis

TMAs obtained from biomax was used for in situ hybridization (ISH) at Advantech Inc. (Hyogo, Japan). Proves against Pn, RNAScope^®^ (cat #409181) or Basescope^®^ (custom probe) from Advanced Cell Diagnostics (Newark, CA, USA) was used according to the manufacture’s instruction. PPIB (positive probe) and DapB (negative prove) were used for the quality check of the tissue section. For acquisition and analysis of single nucleus RNA-seq data, a human single nucleus RNA-seq dataset consisting of 88 031 cells on the Single Cell Portal was used to investigate the expression of Pn across various cell types in PDAC.

### 4.8. Kaplan–Meier Survival Analysis and Single-Cell Sequencing Analysis in PDAC

Using the Kaplan–Meier Ploter database, we investigated the correlation between prognosis and Pn expression in patients with stage I to IV pancreatic cancer. (http://kmplot.com/analysis, accessed on 1 June 2024) The Single Cell Portal was used to perform single nucleotide sequencing of untreated pancreatic cancer. (https://singlecell.broadinstitute.org/single_cell/study/SCP1089/human-treatment-naive-pdac-snuc-seq, accessed on 1 June 2024).

### 4.9. Cell Line-Derived Allogeneic Transplantation Model Studies in Mice

All mouse allograft experiments were approved by the Institutional Animal Committee at the Department of Veterinary Science of Osaka University Graduate School of Medicine (approved number; 04-101-009) and followed the recommendations of the guidelines for animal experimentation at research institutes (Ministry of Education, Culture, Sports, Science and Technology, Japan), guidelines for proper conduct for animal experimentation (Science Council of Japan), guidelines for animal experimentation at institutes (Ministry of Health, Labor and Welfare, Japan) and the ARRIVE guidelines. In all studies, 1.5 × 10^5^ 6422C1 cells (YFP-positive) suspended in 0.1 mL PBS were inoculated on the back of 8-week-old male C57BL6J wild-type mice or Postn-tdTomato lineage tracing mice. Postn-tdTomato lineage tracing mice were established by crossing B6.129S-Postn tm2.1 (cre/Esr1*) Jmol mice and B6. Cg-Gt (ROSA) 26Sortm14 (CAG-tdTomato) Hze/J were obtained from the Jackson laboratory (Bar Harbor, ME, USA). Eight-week-old Postn-tdTomato lineage tracing mice were treated with an intraperitoneal injection of tamoxifen for 5 days before being sacrificed, and the tumors were isolated for the following immunofluorescent study. C57BL6 and periostin-lineage tracing mice were used in this study.

### 4.10. Cell Separation and Fluorescence-Activated Cell Sorting

The excised tumors were minced and then subjected to cell separation using gentle MACS Dissociator (Cat. #130-093-235, Miltenyi Biotec’s, Bergisch Gladbach, Germany), Debris Removal Solution (Cat. #130-109-398), and Red Blood Cell Lysis Solution (Cat. #130-094-183). The separated samples were then sorted using a FACS Aria IIIu (BD bioscience, Milpitas, CA, USA). To remove cells other than CAF, FITC anti-mouse Ep-CAM antibody (Cat. #118207, BioLegend, San Diego, CA, USA), FITC anti-mouse CD45 antibody (Cat. #103108, Invitrogen, Waltham, MA, USA) and FITC anti-mouse CD31 antibody (Cat. #102405, BioLegend) were used as an epithelial cell, blood cell and an endothelial marker, respectively. YFP-positive 6422C1 cells were also included and removed in the FITC-positive cell population. Hematopoietic, epithelial, cancer and endothelial cells were removed by FITC gate using the above-mentioned FITC conjugated antibodies, and the remaining CD90-positive cell population was sorted for RNA-seq using the CAF marker APC anti-mouse CD90.2 antibody (Cat. #17-0902-81, Invitrogen) for CD90-positive/Td-tomato positive and negative cells [33].

### 4.11. Protein Structural Analysis of Pn-ASVs and HSPA1A

NCBI Reference Sequence NP_001273594.1 (Pn 2-1), NP_001129407 (Pn 4-1) and NP_005336 (HSPA1A) were used to search for Pn and HSPA1A protein structure. Phyre2 software (V2.0) ‘Intensive’ modelling mode was used to predict the tertiary structure of each isoform of human Pn and HSPA1A protein. Intensive modelling mode runs a complete modelling of the entire protein tertiary structure using multiple templates and ‘ab initio’ techniques, which could predict structures of the previously unknown protein regions. The structure with the highest confidence rating was initiated with the modelling software Java molecular (Jmol), version 14 which allows for interactivity and visual coding of the structure. Prediction of the 3D structure of a protein complex from the two structural protein data banks (PDBs) files was performed with ClusPro 2.0 docking tool. This tool outputs the top 10 predicted 3D structure models determined by ‘centres of highly populated clusters of low energy docked structures’. The docking model with the highest confidence was selected for each Pn-ASV, and the structures were visualized by PyMol 2.6.

### 4.12. Statistics

For statistical analysis, the values are shown as the means ± SE. All statistical analyses were performed with EZR [34], which is for R. For statistical analysis of expression change of two groups, an unpaired *t*-test for normal distribution data and Mann–Whitney U test for non-normal distribution data were performed. A one-way ANOVA test for multiple comparisons and post-hoc Tukey HSD for pairwise comparison were used. A *p*-value less than 0.05 was considered as statistically significant.

## 5. Conclusions

Pn is a key ECM protein in various tumor progressions including PDAC. Previously, we described role of Pn-ASVs with specific functional features in breast cancer. In this study, we focused on physiological and pathological Pn-ASVs, and examined the characteristics of Pn-expressing cells and the difference in function of each ASV in PDAC. We demonstrated that (1): CAFs is a main source of Pn synthesis, and selectively secrete pathological Pn-ASVs with exon 21 both in mouse and human specimens. (2): RNA sequencing identified the gene signature of Pn-positive CAFs associated with ECM-related genes and chemokines, factors that shape the chemoresistance TME. (3): Only pathological Pn-ASVs interacted with HSP70-1a, leading to significant rescue of gemcitabine-induced PDAC apoptosis. (4): In silico analysis revealed that the presence or absence of exon 21 changes the tertiary structure of Pn and the binding sites for HSP70-1a. These findings indicated that Pn-ASVs with exon 21 secreted from CAFs play a crucial role in supporting tumor growth by interacting cancer cell-derived HSP70-1a, suggesting that Pn-ASVs with exon 21 might be a potential therapeutic and diagnostic target in PDAC patients with rich stroma.

## Figures and Tables

**Table 1:** Primer list.

Gene Name	Forward	Reverse
*Mouse Pn 1*	ATAACCAAAGTCGTGGAACC	TGTCTCCCTGAAGCAGTCTT
*Mouse Pn 2*	CCATGACTGTCTATAGACCTG	TGTCTCCCTGAAGCAGTCTT
*Mouse Pn 3*	ATAACCAAAGTCGTGGAACC	TTTGCAGGTGTGTCTTTTTG
*Mouse Pn 4*	CCCCATGACTGTCTATAGACC	TTCTTTGCAGGTGTGTCTTTT
*Mouse Pn exon 9–10*	CAGCAAACCACTTTCACCGACC	AGAAGGCGTTGGTCCATGCTCA
*Mouse Col 1a1*	TTCTCCTGGCAAAGACGGAC	CGGCCACCATCTTGAGACTT
*Mouse Fbn1*	GTGGCTGTGAAAGGGAACCA	TGAAGCCAATCCTTGGAGCG
*Mouse Itgb3*	GTGAGTGCGATGACTTCTCCTG	CAGGTGTCAGTGCGTGTAGTAC
*Mouse Itgb5*	CTTACCCTGGTCAGAGGAAGTG	CCTCAAGGTGAAAGACTGTGCTG
*Mouse Tgfb1*	GTGGAAATCAACGGGATCAG	ACTTCCAACCCAGGTCCTTC
*Mouse Ctgf*	AGGGCCTCTTCTGCGATTTC	CTTTGGAAGGACTCACCGCT
*Mouse MMP2*	CCCTAAGCTCATCGCAGACT	GGCTGCTTCACATCCTTCAC
*Mouse MMP9*	CTTTGCTTCAACCGTGCAGT	AAAAGGTTGGGGATCCGTGT
*Mouse Fzd1*	ACAAACAGCACAGGTTCTGC	GCTCCAAATACGTAAATTGCCC
*Mouse Ccl2*	GCTACAAGAGGATCACCAGCAG	GTCTGGACCCATTCCTTCTTGG
*Mouse 18s rRNA*	ATGGCCGTTCTTAGTTGGTG	CGGACATCTAAGGGCATCAC
*Human Pn exon 10–11*	TACAACGGGCAAATACTGGA	CTTGATGATCTCGCGGAATA
*Human Pn exon 16–17*	TGTTCGTGGTAGCACCTTCAA	TGATAATAGGCTGAAGACTGCC
*Human Pn exon 21–22*	GGTCACCAAGGTCACCAAATTC	TGTTGGCTTGCAACTTCCTCAC
*Human 18s rRNA*	CGGCTACCACATCCAAGGAA	CTGGAATTACCGCGGCT

## Data Availability

The data presented in this study are available on request from the corresponding author.

## Supplementary Materials

The following supporting information can be downloaded at: https://www.mdpi.com/article/10.3390/ijms252313205/s1.
