# Supplementary material for: A Role for Periostin Pathological Variants and Their Interaction with HSP70-1a in Promoting Pancreatic Cancer Progression and Chemoresistance
Source: Int J Mol Sci. 2024 Dec 8;25(23):13205. doi: 10.3390/ijms252313205 (PMC11641934; doi:10.3390/ijms252313205)
Supplement: Supplementary file 1 [file ijms-25-13205-s001.zip › ijms-3312112-supplementary.pdf]

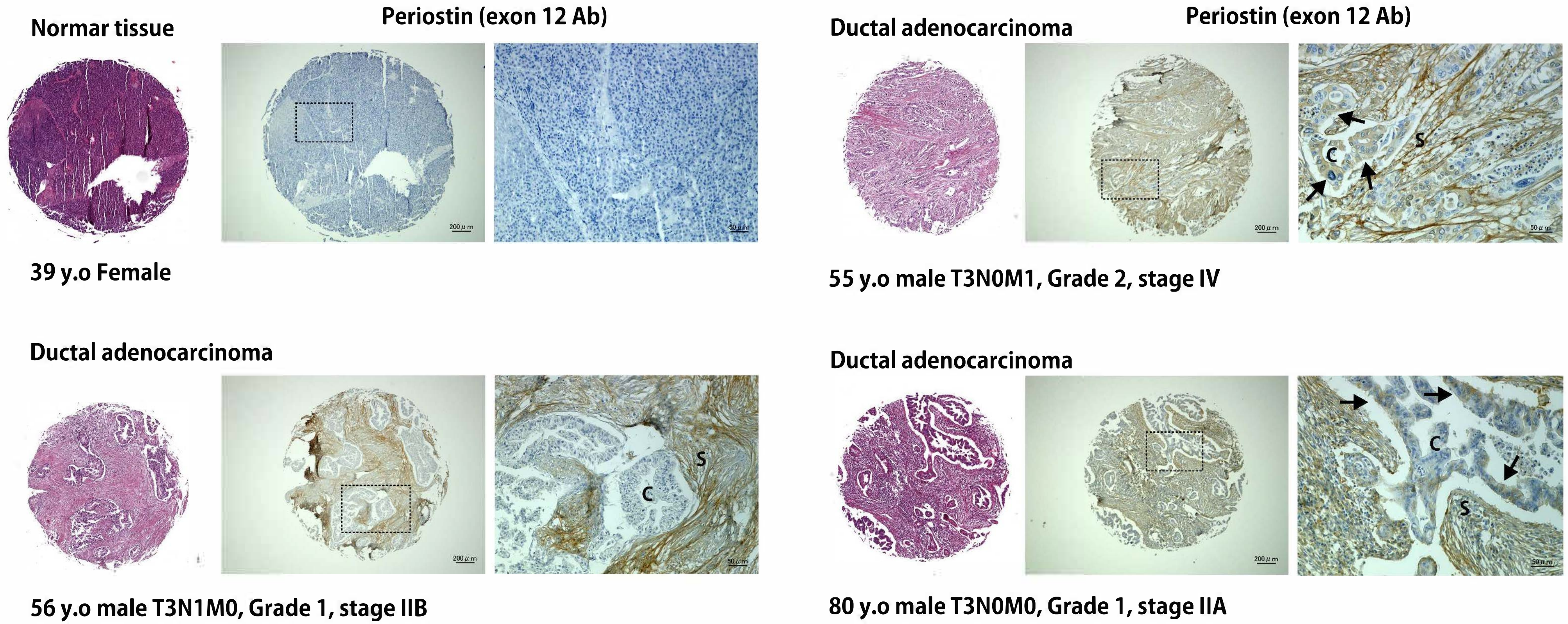

Supplement figure S1

Representative Pn immunohistochemical staining of normal pancreas and PDAC. Arrows indicate the positive staining in cancer parenchymal cells in PDAC, although most of positive signals were observed in cancer stroma.

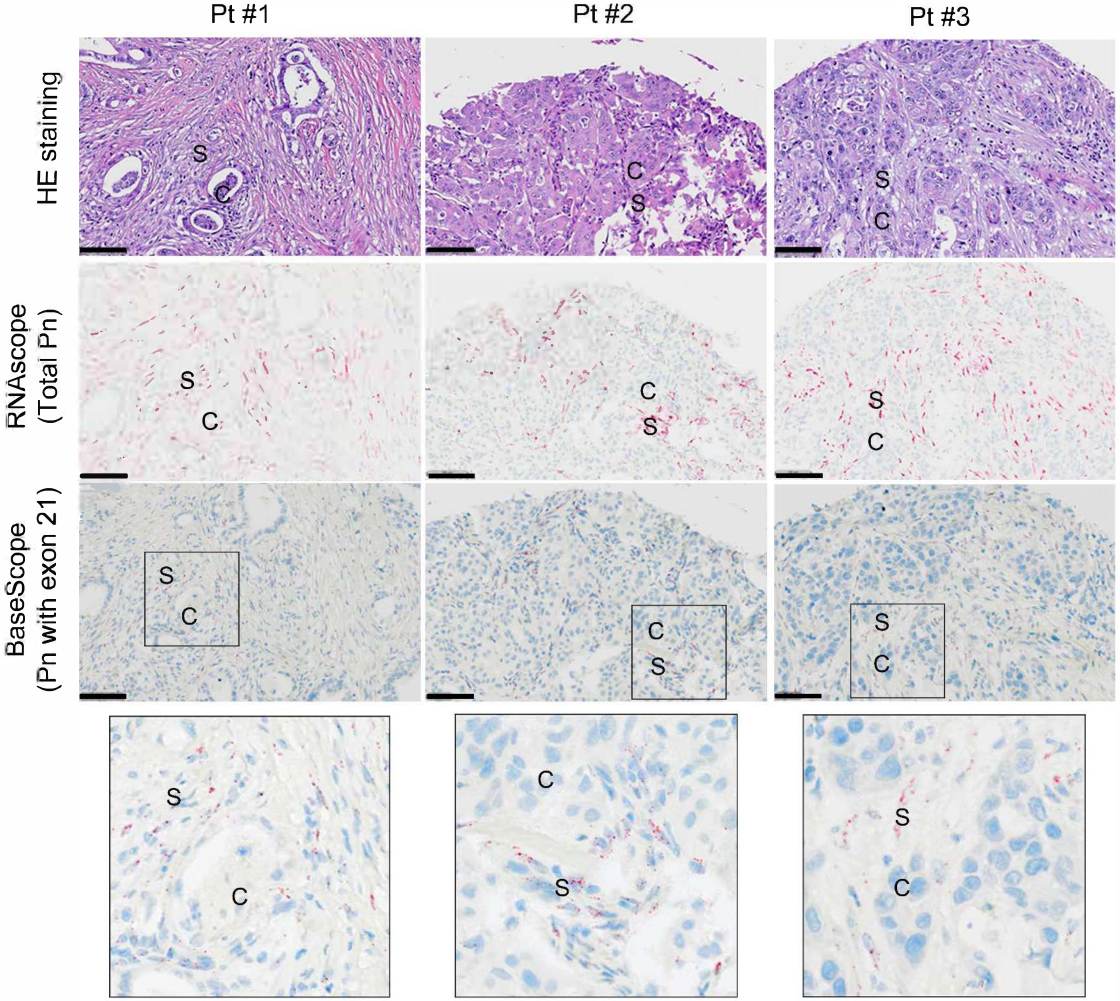

	Age	Sex	Pathology diagnosis	TNM	Grade	Stage
Pt #1	72	F	Duct adenocarcinoma	T3N0M0	1	IIA
Pt #2	44	M	Duct adenocarcinoma	T3N0M0	3	IIA
Pt #3	78	M	Duct adenocarcinoma	T3N0M0	3	IB

Supplement figure S2

Three representative in situ hybridization images of total Pn (RNAscope) and Pn-ASVs with ex-on 21 (Basescope) in pancreatic cancer specimens.
The red color indicates a positive signal for Pn mRNA. Both total Pn and Pn-ASVs with exon 21 mRNA was expressed in stroma surrounding cancer cells.

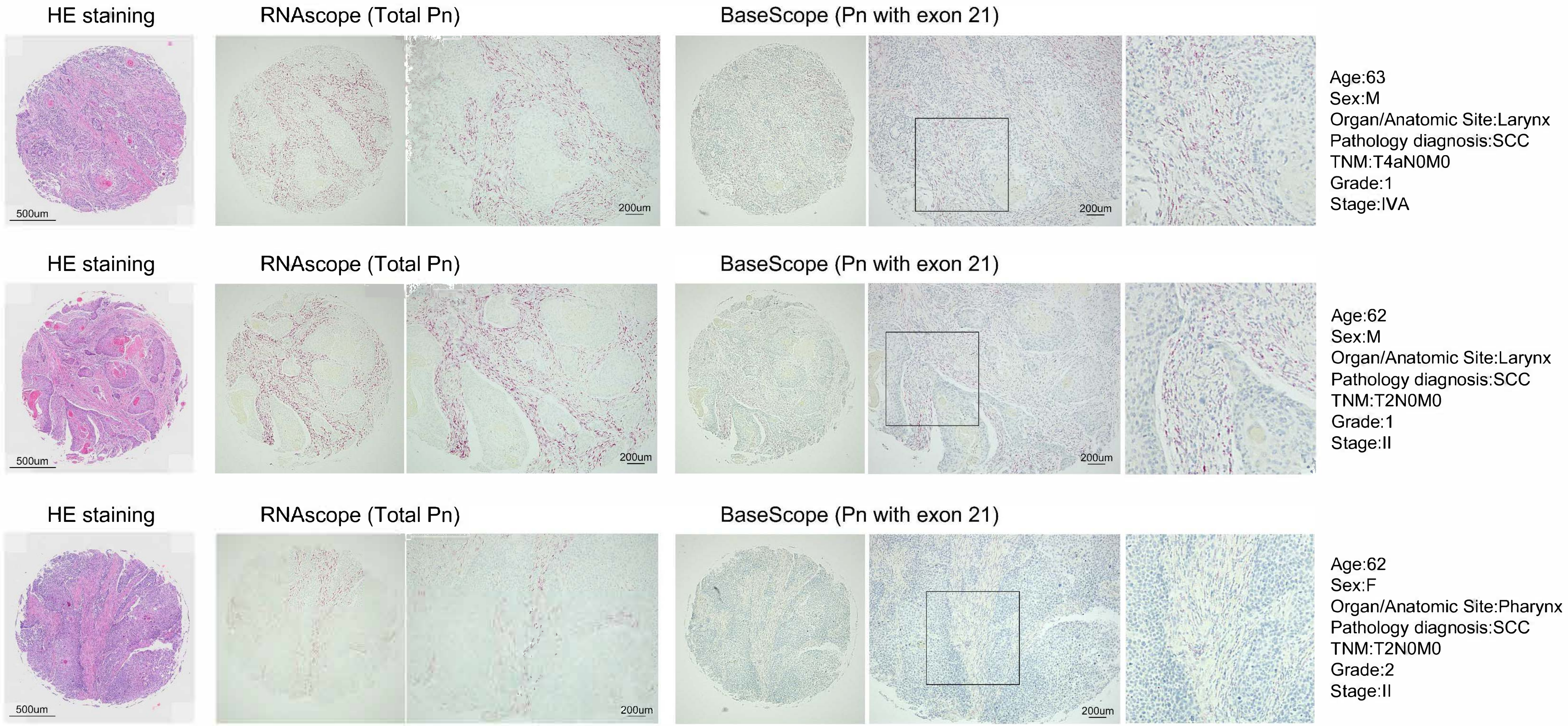

Supplement figure S3

Three representative in situ hybridization images of total Pn (RNAscope) and Pn-ASVs with ex-on 21 (Basescope) in head and neck cancer specimens. The red color indicates a positive signal for Pn mRNA. Both total Pn and Pn-ASVs with exon 21 mRNA was expressed in stroma surrounding cancer cells.

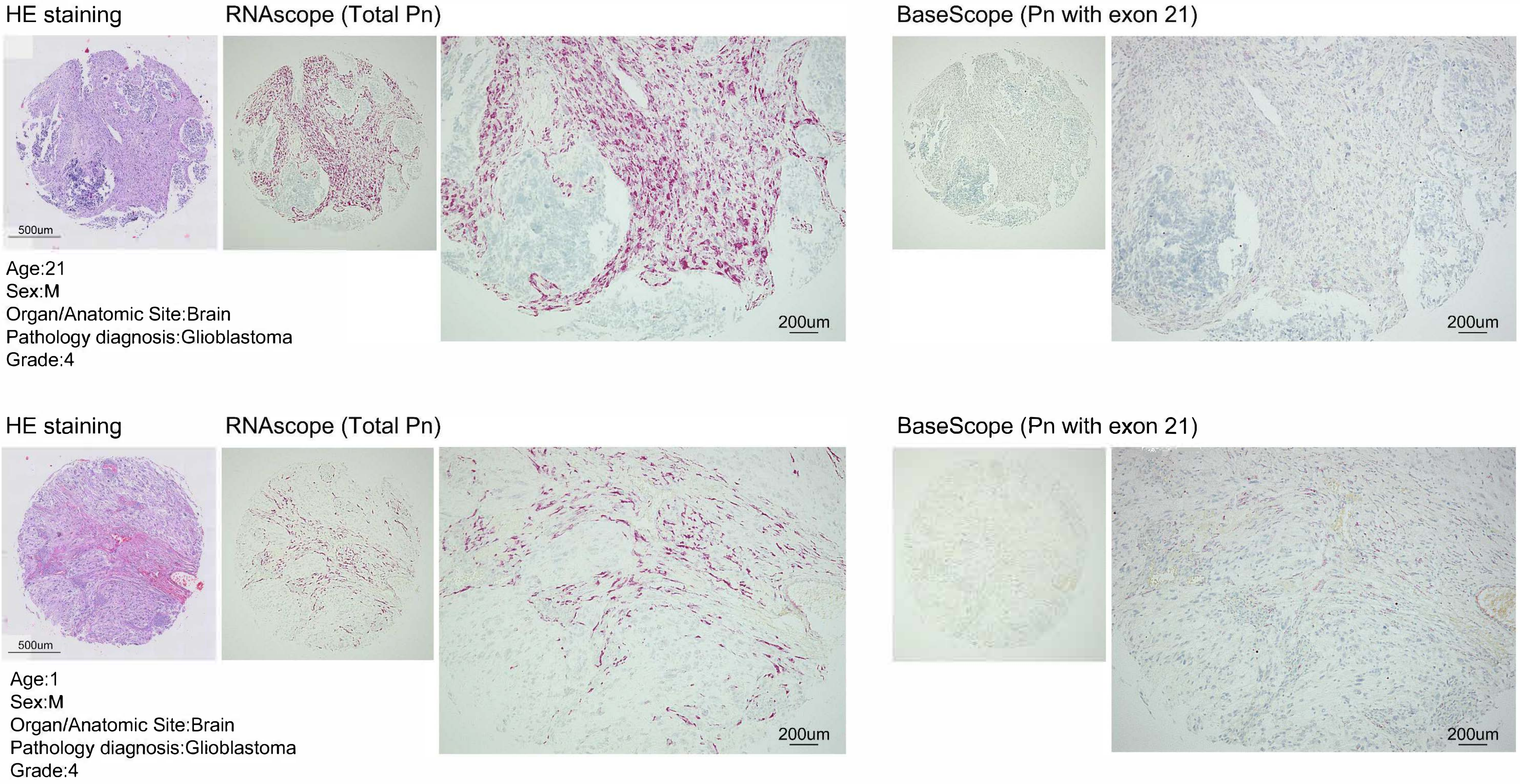

Supplement figure S4

Representative in situ hybridization images of total Pn (RNAscope) and Pn-ASVs with exon 21 (Basescope) in glioblastoma specimens.
The red color indicates a positive signal for Pn mRNA. Both total Pn and Pn-ASVs with exon 21 mRNA was expressed in stroma surrounding cancer cells.

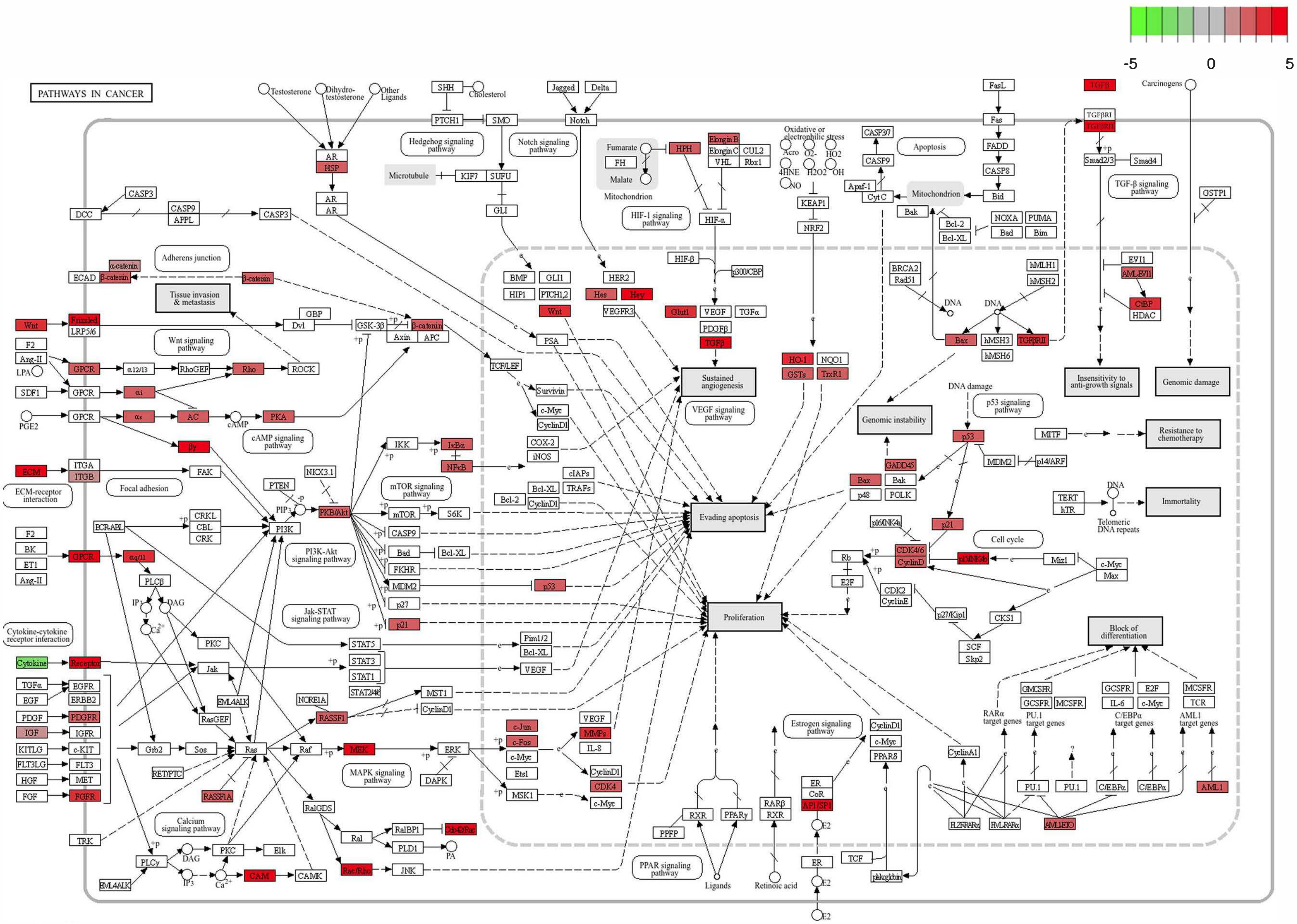

Supplement figure S5

Pathway in cancer; red indicates up-regulated genes and green indicates down-regulated genes in Pn-positive CAFs compared to Pn-negative CAFs.

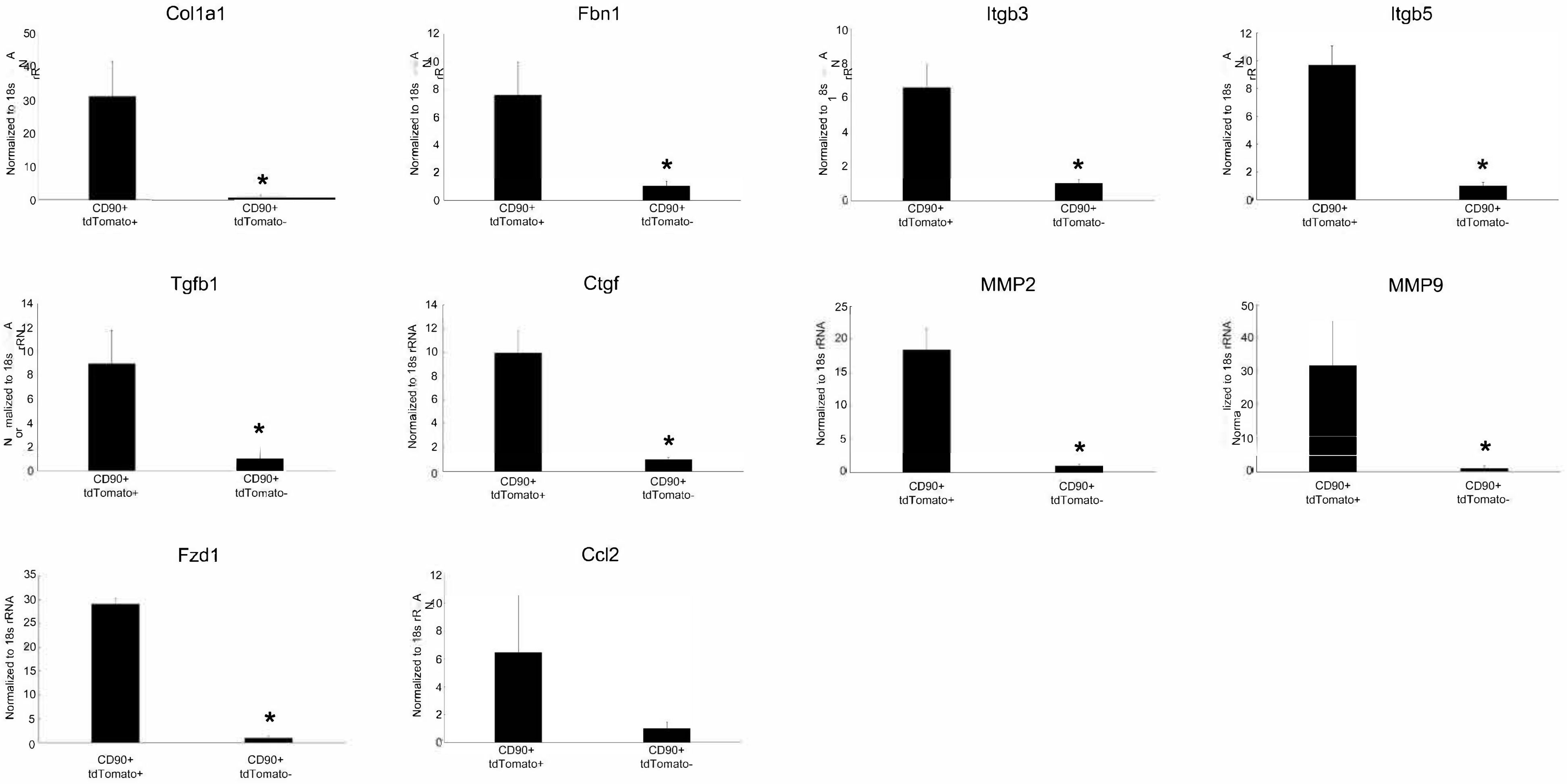

Supplement figure S6

Relative expression pattern analysis of upregulated genes in Pn-positive (CD90+tdTomato+) and –negative (CD90+tdTomato-) CAFs by qRT-PCR analysis to validate the RNA-seq data shown in figure 5. N=3, *$p<0.05$ vs Pn-positive CAFs.

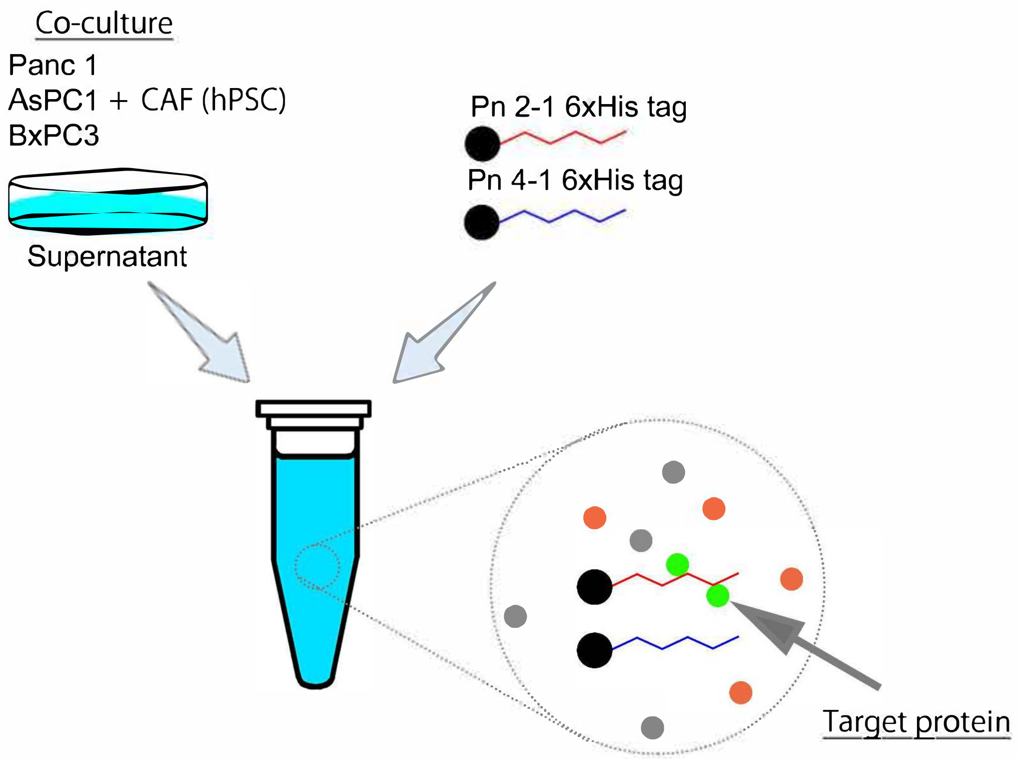

Supplement figure S7

His pulldown assay was performed using His-tagged recombinant Pn and co-culture super-natants of PDAC cell line and CAF cell line to search for proteins that interact with Pn-ASVs with exon 21 (Pn2-1) or Pn-ASVs without exon 21 (Pn4-1).
